# Revisiting bioethics in the Indian context: A review

**DOI:** 10.6026/973206300220538

**Published:** 2026-01-31

**Authors:** Jhanvi Vaghela, Sunil M Doshi

**Affiliations:** 1 Department of Pharmacology, Dr. N.D. Desai Faculty of Medical Science and Research, Dharmsinh Desai University, Nadiad, Gujarat, India; 2 Department of Forensic Medicine, Dr. N.D. Desai Faculty of Medical Science and Research, Dharmsinh Desai University, Nadiad, Gujarat, India

**Keywords:** Bioethics, AETCOM, autonomy, beneficence, non-maleficence, justice

## Abstract

In the present era, where cases of medical negligence and issues pertaining to ethical breaches in the doctor-patient relationship are on the rise, revisiting "not so modern" terms and theories encircling bioethics in medical practice is imminent. National Medical Commission has incorporated Bioethics within AETCOM (Attitude, Ethics, and Communication) and it has become an integral part of the medical curriculum with specific allocated module along with methods of assessment. However, it is observed that not much emphasis is being given to AETCOM modules and hence attitude and perception of the students and the teachers need to shift from "ignorance to importance" for the benefit of the future physician-patient relationship.

## Background:

**Figure 1:**
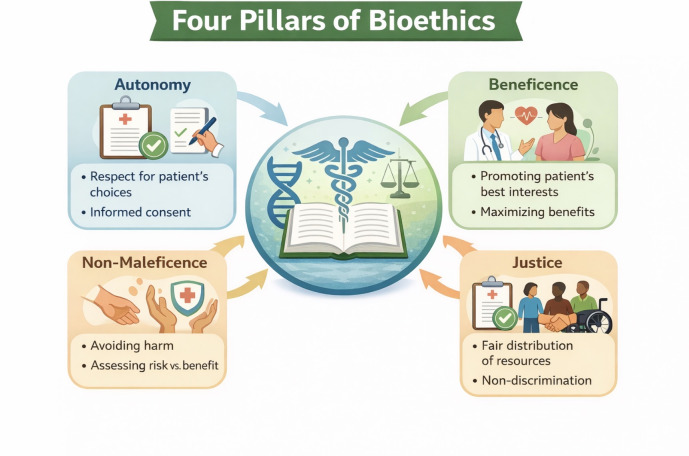
Depiction of four pillars of bioethics

Ethics, in a philosophical way, arises from a question: what makes honest actions right and dishonest ones wrong? [1]. Ethics is a broad field that seeks to understand concepts of right vs wrong while dealing with moral principles set within society. Bioethics is a field within this broad term that focuses on ethical issues related to biology. It is a philosophical discipline surrounding social, legal, cultural, epidemiological, and ethical issues arising out of development in healthcare and life science research [2]. The term, if broken, is divided into Greek bios, meaning life, and ethos, meaning moral behaviour. Bioethics refers to the ethical issues arising from healthcare and the biomedical sciences. Medical ethics, although often cited as it had its beginning in the days of Hippocrates in ancient Greece, is in fact much older than that [3]. Philosopher Fritz Jahr, in 1927, authored "Bio-Ethics: A Review of the Ethical Relationships of Humans to Animals and Plants," proposing a "Bioethical Imperative" that broadened Kant's moral principle to include every form of life [4]. There are four pillars of biomedical ethics, quoted by Beauchamp *et al.* in their book, as follows: autonomy (a norm of respecting and supporting autonomous decisions), beneficence (a group of norms pertaining to relieving, lessening, or preventing harm and providing benefits and balancing benefits against risks and costs), non-maleficence (a norm of avoiding the causation of harm) and justice (a group of norms for fairly distributing benefits, risks, and costs) [5]. Figure 1 illustrates the four pillars of bioethics.

## Autonomy:

Autonomy is the first and most vital principle out of the four pillars of bioethics. Respecting individual autonomy is the foundation of democratic thinking [6]. Prior to the establishment of bioethics, discussions of autonomy were limited within twentieth-century philosophy and seldom appeared in the context of healthcare ethics [7]. Within the ambit of modern medical practice, it accentuates an individual's right to make decisions related to his/her own health. After being adequately summarized about the overall condition of health as well as pros and cons about further interventions to be taken into account, the patient has the right to fully accept, partially accept, or even reject the expected plan. In short, the patient should not be taken for granted in terms of his/her healthcare. A study has concluded that confidence and trust in healthcare providers and treatment with respect and dignity are more closely associated with patients' overall evaluations of their hospitals than adequate involvement in the healthcare decisions [8].

## Beneficence:

Beneficence is "doing well" always keeping in mind to choose the best for the particular patient. Synonyms of the term are benevolence, kindness, generosity, compassion, etc. Acts of beneficence exclude those performed accidentally or without the intent to promote another's welfare. For example, saving a patient's life may not constitute mercy, nor can it be simply termed charitable; it reflects the basic moral obligation inherent in medical practice [9]. Taking proactive steps keeping the benefit of the patient in mind is beneficence. Although the term is applied broadly in ordinary discourse, ethical theory interprets beneficence in an even more comprehensive manner that encompassing all moral norms, dispositions, and actions that seek to enhance or promote the well-being of others [10]. Any intervention in terms of providing healthcare should be evidence-based and outweigh potential risks. Healthcare professionals are expected to develop and sustain their competencies, engage in continuous learning, take into account the unique circumstances of each patient, and consistently act in ways that promote patients' welfare and overall benefit [11]. It incorporates the core value of "being a doctor" and serving the sufferers. To act in the best interests of the patient, both the physician and the patient must arrive at a shared understanding of what constitutes the patient's good. Such understanding can only be achieved through open dialogue between the physician and the patient and/or family with acknowledgement of the patient's autonomy, self-perception, and freedom to choose among available treatment options [12]. In the context of research, it is applicable to designing the study with a favourable risk-benefit ratio. Offering a novel therapy with expected benefits and better health outcomes should outweigh the risk involved while considering the study's contribution to the benefit of society in a clinical trial.

## Non-maleficence:

Non-maleficence is the principle of bioethics with the literal meaning "does no harm" and is derived from the maxim primum non nocere, meaning "first, do no harm" [13]. Beneficence refers to the moral responsibility to act in ways that promote good and prevent harm, while non-maleficence emphasizes the duty to refrain from causing injury or suffering. For instance, deliberately pushing someone down for laughter clearly violates non-maleficence, whereas assisting a person who has fallen reflects beneficence [14]. A physician is expected to avoid causing possible harms either through acts of omission or acts of commission. Almost every medical decision involves some degree of balancing between beneficence and non- maleficence. Yet, evaluating the potential benefits and risks in such situations is rarely a simple or straightforward task [15]. It can be considered the other side of a coin with beneficence on one side. If a treatment is expected to cause more harm than benefit, even if evidence states that it's the best available choice, one may avoid it in that particular scenario, looking into the long-term overall benefit of the seeker. In day-to-day clinical practice, especially in India, non-maleficence is sometimes a neglected rule of medical bioethics when dealing with "rush." There has been a persistent shortage and inequitable distribution of human resources in India over the years, with the rural posts experiencing the most shortage [16].

## Justice:

Justice is generally understood as fair, equitable, and appropriate treatment of persons [17]. While doing medical practice, a physician is expected to become fair or impartial to their patients. Simply giving priority to patients during OPDs based on relation, social status, peer pressure, caste, or religion is "unfair" and fails to follow the fourth pillar of medical bioethics. The three most important aspects under the umbrella of "justice" are rights-based justice, distributive justice, and legal justice [18]. Respecting patients' rights i.e. to be consulted properly, to be treated equally, and not to be discriminated against, should be an integral part of practice. Every citizen, being a patient, has a right to be treated equitably. Justice demands that limited resources be distributed fairly and those patients not be favoured or discriminated against due to race, religion, gender identity, sexual orientation, age, or cultural background [19]. As far as "distributive justice" is concerned, this applies to the distribution of limited healthcare resources among the needy population. If we take the example of the COVID-19 vaccine, the earlier distribution of limited doses was among those who were more vulnerable to infections and candidates for expected fatal outcomes due to personal health. Due to high demand and low production in an unprecedented situation like the COVID-19 pandemic, the distribution became difficult or sometimes impossible [20]. Every physician is expected to observe not only ethical standards under "codes of conduct" but also to follow rules laid down under the law of the land and that reflects to legal justice.

## Bioethics in Indian medical education:

There is no better way than making "Bioethics" a part of the medical undergraduate curriculum. A welcome step was taken by the National Medical Commission to incorporate Bioethics within AETCOM (Attitude, Ethics, and Communication) [21]. Earlier, in the absence of AETCOM modules, bioethics was learned by students through experience and observing consultants during clinics. But now, when it has become an integral part of the curriculum, the student is expected to learn these core aspects thoroughly and formally, including being assessed for the same. Even under OSPE/OSCE, students are assessed in a way that incorporates the empathetic aspect while dealing with patients during examinations. Their communication with patients is observed and judged, rather than limiting the examination to inspection, palpation, percussion, and auscultation. However, it is observed that not much emphasis is being given to AETCOM modules at many institutes [22]. The attitude and perception of students and teachers need to shift from "ignorance to importance" for the benefit of the future physician-patient relationship.

## Conclusion:

Bioethics is often discussed in philosophical language in articles and books. However, its essence lies in guiding clinicians to think beyond the technical aspects of their clinical practice and to approach patients with empathy and understanding. Medical negligence cases are rising day by day, not only because of ignorance or incompetence but also as a result of ignoring these four pillars of medical bioethics. It should be embraced as a professional duty rather than merely an obligation.
